# Establishing Australia’s national linked COVID Register

**DOI:** 10.23889/ijpds.v9i5.2807

**Published:** 2024-09-18

**Authors:** Robert Goerge

**Affiliations:** 1 Chapin Hall

To link social program data, such as employment, welfare, education, and human services data, a set of high-quality variables or fields are needed to facilitate rigorous linkage. The siloed nature of data collection in the US leads to a lack of consistency in the data that agencies collect. The data that one needs to make high quality linkages are often exactly those that government agencies are unwilling and unable to collect, or those that have poor quality. This has significant implications for privacy-protecting record-linkage and other techniques whereby privacy and confidentiality can be appropriately addressed. There are some obvious examples. For example, in the US, many agencies are unlikely to collect or share Social Security Number because of privacy concerns. A different type of example many agencies collect race or ethnicity poorly, so that the data is not consistent over time or simply inaccurate at the point of collection. Some programs may only collect age or month and year of birthdate. This presentation will discuss strategies to address these barriers short of changing agency practice around data sharing. It will also address how policies are a barrier to better linkage and data use. Also, how the proposed National Secure Data Service may affect state capacity to conduct linkage will also be discussed. This presentation is based on 40 years of experience working with the US federal government, states, and local agencies.

